# Concurrence of myotonic dystrophy and epilepsy: a case report

**DOI:** 10.1186/1752-1947-8-427

**Published:** 2014-12-15

**Authors:** Dawit Kibru Worku

**Affiliations:** Department of Neurology, Addis Ababa University, Addis Ababa, 29818 Ethiopia

**Keywords:** Clinical hand grip, Epilepsy, Myotonic dystrophy, Percussion myotonia

## Abstract

**Introduction:**

Myotonic dystrophy is a clinically and genetically heterogeneous multisystem disorder with a prevalence of 1 in 8000 in the general population.

**Case presentation:**

A 25-year-old Ethiopian man presented with symptoms of myotonia, muscle wasting, gait problems, frontal baldness, and family history characterizing the hereditary disorder myotonic dystrophy. He had been on treatment for idiopathic generalized epilepsy for over 15 years. A needle electromyography showed insertional classic myotonic discharges. A nerve conduction study showed mild axonal sensorimotor polyneuropathy. His muscle biopsy showed marked increase of internalized nuclei, severely atrophic muscle fibers, muscle fiber necrosis and regeneration of isolated muscle fibers, architectural changes, and a preferential atrophy of type I fibers.

**Conclusion:**

This is a rare occurrence of two distinctive hereditary diseases.

## Introduction

Myotonic dystrophy (DM) is a clinically and genetically heterogeneous multisystem disorder with a prevalence of 1 in 8000 in the general population [1]. Individuals with DM may show neuromuscular and cognitive dysfunctions, hypersomnolence, cataracts, cardiac conduction abnormalities, infertility, and insulin resistance [2]. The association of DM and epilepsy is rare and infrequent. In affected persons, mental retardation is a frequent complication [2]. This case presents a very rare coexistence of generalized epilepsy in a patient with DM type-I, and questions the possibility for a shared pathophysiologic genetic precursor.

## Case presentation

A 25-year-old right-handed Ethiopian man presented with a 2-year history of generalized weakness and wasting of his face, hands and feet. He often complained of stiffness and had difficulty in releasing his grip, as following a hand shake. He also has slow and difficult swallowing, and excessive daytime sleepiness. There was no history of fever, neck stiffness, ear discharge, or trauma. He had been on treatment for idiopathic generalized epilepsy (IGE) for over 15 years, and was admitted for an episode of severe pneumonia in the preceding month. His family history is positive for DM in his mother and younger brother, and IGE in his elder brother. There was no past history of diabetes, hypertension, pulmonary tuberculosis, syphilis, alcohol or tobacco intake, or other substance abuse.

### Examination

He was thin built and without anemia, jaundice, or cyanosis. His cortical functions were normal. His speech was nasal. His face was long and narrow with hollowed cheeks and sagging jaw, and frontal baldness. His masseter, pterygoid and temporalis muscles were weak bilaterally. He had no cataract. Intrinsic muscles of his hand, muscles of his distal forearm, and ankle dorsiflexors were moderately weak and atrophied on both sides. There were clinical hand grip and percussion myotonia. His superficial and muscle stretch reflexes were normal. His primary and cortical sensations were normal. His gait was high stepping. The result of examination of his other system organs was normal.

### Investigations

The results of his routine hematologic and chemistry laboratory tests were normal. His serum fasting blood glucose, follicle-stimulating hormone and luteinizing hormone levels were normal. His serum muscle enzymes and a routine electroencephalogram were normal. A needle electromyography showed insertional classic myotonic discharges. A nerve conduction study showed mild axonal sensorimotor polyneuropathy.

**Figure 1:**
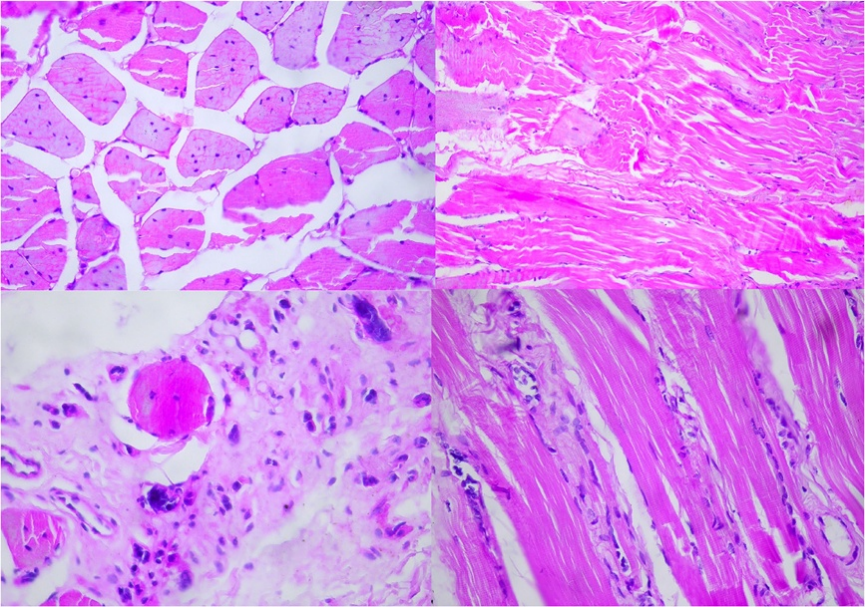
**Muscle biopsy showed marked increase of internalized nuclei, severely atrophic muscle fibers, muscle fiber necrosis and regeneration of isolated muscle fibers.**

A muscle biopsy showed marked increase of internalized nuclei (arrayed in chains in longitudinal section), severely atrophic muscle fibers with pyknotic nuclear clumps, muscle fiber necrosis and regeneration of isolated muscle fibers, architectural changes such as sarcoplasmic masses and ring fibers, and a preferential atrophy of type I fibers (Figure 1).

He was subsequently discharged after he improved after pneumonia and his seizures were controlled with 300mg of phenytoin per day.

## Discussion

This patient presented with symptoms of myotonia, muscle wasting, gait problems, frontal baldness, and family history characterizing the hereditary disorder DM. In most studies on the myopathies, attention has been focused on muscle tissue per se. It is only recently that the central nervous system, the blood serum, the cardiovascular system, and other areas have come under scrutiny. Electroencephalograms done on 101 patients with muscular dystrophy and related myopathies showed abnormalities in 39%.

Simultaneous occurrence of DM and epilepsy has been rarely reported. In one case report from India a patient had all the classical features of DM and associated generalized epilepsy [1]. In another case report a patient had clinical characteristics and findings of both DM and progressive myoclonus epilepsy of the Unverricht–Lundborg type [2]. Our patient, in addition to the classic features of DM and IGE has positive family history for both diseases.

Despite having two different progressive inherited disorders affecting the central nervous system, the patient showed only mild mental retardation or none with very slow progression. Dementia has been reported in some cases which was not the case in our patient [3, 4]. In the future, detailed clinical screening for dementia and mental retardation, and frequent electroencephalogram studies may point to the involvement of the central nervous system in DM.

The major difference we notice between our case and reports in other parts of the world is that impaired mental functions are common in other reports, but not in our patient [5]. In addition, the type of seizure associated was IGE in our patient whereas progressive myoclonus epilepsy of the Unverricht–Lundborg type was present in the other report [1]. In the future we anticipate more differences could be drawn as the number of cases reported increase.

## Conclusions

This is a rare occurrence of two distinctive hereditary diseases. Our patient had all the classical features of DM. He had associated generalized epilepsy. The case showed the possibility for a shared pathophysiologic genetic precursor for the two diseases. Further studies are needed to confirm this finding, and to further increase our understanding of this association.

## Patient’s perspective

I was told I had epilepsy while I was a child and was taking the medications. When I started to have weakness in my hand I thought the antiepileptic drugs caused this. But my doctors told me it was not related with the epilepsy and referred me to a neurology center. In the neurology unit they told me to have a muscle biopsy and nerve conduction test, and some others too. After the result they told me I have a muscle disease which is progressive.

I have difficulty in running, walking on uneven ground and most of all I couldn’t go to school because the school is far and I have to walk a long distance. I was having repeated pneumonia and the doctors told me it may be related to my muscle disease. The most devastating thing to hear is that there is no cure. Since I start follow up at the neurology unit even though I am seizure free the weakness is progressing.

I am thankful for my doctors and I want to remain anonymous.

## Consent

Written informed consent was obtained from our patient for the publication of this case report and any accompanying images. A copy of the written consent is available for review by the Editor-in-Chief of this journal.

## Author’s information

Dawit Kibru Worku, MD is a senior medical specialist (Neurologist) currently works as a University lecturer and clinician, Addis Ababa, Ethiopia.

## Abbreviations

DM: Myotonic dystrophy
IGE: Idiopathic generalized epilepsy.
